# Making un/equal: reassessing inequality and mental health through a praxeographic approach on welfare categorization processes

**DOI:** 10.1007/s00127-023-02550-9

**Published:** 2023-09-16

**Authors:** Milena D. Bister

**Affiliations:** Institute of European Ethnology, Humboldt-Universität zu Berlin, Berlin, Germany

**Keywords:** Mental health, Social inequality, Welfare classification practices, Social policy, Praxeography, Disability

## Abstract

**Purpose:**

In recent decades, Europe has seen a steady increase in psychiatric diagnoses, which, besides affecting the population in many ways, also challenges the organization of welfare. This paper explores how welfare classification processes impact the contemporary production of mental (ill) health and social inequality in the German welfare state.

**Methods:**

Based on comprehensive ethnographic research in the public mental healthcare landscape in Berlin between 2011 and 2017, this paper discusses in detail the case of a mandatory prescription of a psychosocial rehabilitation measure for Ms Reisch, a psychiatric service user and ethnographic research partner. The analysis draws on the methodological approach of praxeography to examine how this case challenges the social determinants of mental health framework and the conceptual work of the sociology of inequality on which the categories of welfare are largely built.

**Results:**

The paper highlights the essentializing properties of social categories, whether in the sociology of inequality or in social and mental health policy. It also demonstrates the strength of praxeography to expose how multiple welfare categorization processes shape experiences and events of dis/ability in practice, potentially contradicting the stated intentions of social policy.

**Conclusion:**

The results suggest that the attachment of categories *to people* in public welfare needs to be changed to make public administration more flexible to responding to the situated processes that bring about differentiations of equal and unequal in practice. The paper, therefore, encourages social inquiry into the potentialities of a post-categorical social policy framework.

## Introduction

During my ethnographic research on knowledge practices in German psychiatric hospitals and the mental healthcare sector, I noted that, to resume everyday life and to plan healthcare interventions, clients and staff often referred to social conditions. Family status, employment, housing, asylum status, education, age, or gender featured prominently as aspects of what was considered the ‘social’. It struck me that these references allowed for two contradictory conclusions: mental health afflictions were considered to be embedded in a larger social context, but, at the same time, the person afflicted seemed to remain responsible for coping individually with this broader context in their own lives, albeit with the support of the welfare state. This is similar to what can be observed in the social determinants of mental health framework [1]: it identifies a number of social factors as causes of the unequal distribution of mental health risks in societies, such as racial discrimination, adverse early life experiences, poor education, unemployment, and poverty, but studies that employ the framework rarely take these into consideration in detail when suggesting structural reforms of public policies that prioritize health [2]. In the social determinants of mental health framework, the ‘social’ thus remains a conglomerate of social variables, and their correlations, to which individuals can be, and, in fact, are, ascribed. This rather static understanding of the social as assignable boxes of individual characteristics is also visible in the architecture of the German welfare state, where social policy defines categories of beneficiaries to counteract social inequalities at the level of (entitled) persons [3].

In this paper, to rethink social policy, its connections with social theory, and its impact on social inequality, I start from a different understanding of the social. Based on praxeography and material-semiotic approaches within the field of science and technology studies, I consider it as a process in which phenomena seen as mental (ill) health and inequality are mutually produced, known, and experienced [4, 5]. In this account, rather than complementing the ‘biological’, the ‘psychological’ or the ‘technological’ that have been postulated as independent domains in the history of Western science, the ‘social’ is the process that creates agency, knowledge and experience, so everything that contributes to the assemblage of “matters of concern” [6]. In a nutshell, this paper defines the ‘social’ as all that makes mental health and inequality matter in certain practices. This definition allows us to explore how mental health and inequality acquire meaning in welfare practices, what that meaning is, and why that meaning matters for society.^1^ My aim is to demonstrate that, to develop new forms of shared public accountability for reducing social inequalities and re-imagine social services for a more equitable future, engagement with social theory is necessary.

In what follows, I begin by sharing ethnographically generated details that prompted me to take a closer look at the relationship between mental health and inequality in the German welfare system. The analysis developed here is part of ethnographic research in which I have investigated public mental healthcare practices in Berlin between 2011 and 2017.^2^ Part of this research was recurrent participant observation in the psychiatric departments of two general district hospitals (a total of about 80 days, plus participation in 16 psychiatric home treatments) and four community mental healthcare centers in Berlin (a total of about 30 days), as well as regular meetings outside formal care infrastructures with four people whom I knew as patients from my fieldwork, over a period of 2 years. Central to my research on this topic was the experience of Ms Reisch,^3^ a psychiatric service user and one of my ethnographic research partners, which culminated in a very specific case of a compulsory prescription for psychosocial rehabilitation with serious negative effects on Ms Reisch’s health. To analytically unpack the situation which Ms Reisch became part of, I revisit the conceptual work of the sociology of inequality, on which central welfare categories largely rest, and extend it by introducing a praxeographic approach that builds on the research program of un/doing differences [8]. I thus show how engagement with social theory is important for understanding how categories of welfare are implied in shaping mental health and inequality. Finally, I conclude by proposing a link between praxeography and post-structuralist legal studies, and steps toward an empirically grounded and theory-rich post-categorical reinvention of social policy based on changing welfare categories and who and what they are aimed at.

### “Tough as nails”

When I first met her in 2011, Ms Reisch, a woman in her early 50s and single parent of one child, was a psychiatric inpatient for the first time in her life. Her declared goal was to resume work and take back her child, who had been temporarily placed with a foster family. Over the following 2 years, I regularly visited Ms Reisch in her apartment. We talked about everyday life, ups and downs, her current psychological treatment plans, including therapies and drugs, their limits and effects. With the support of the social worker at the hospital, Ms Reisch applied to the local court for statutory care. She was assigned a guardian who was authorized to sign on her behalf for matters relating to finances, administrative formalities (*Behördenwege*), mail, and health for a limited period of a few years.

During one of our encounters in 2013, Ms Reisch shared very significant news. Her guardian had, without consulting her, signed an agreement for a medical rehabilitation stay proposed by the public Employment Office (*Arbeitsamt*). Being in principle in favor of rehabilitation measures, this signed agreement not only came out of the blue for Ms Reisch, it put her under considerable existential pressure. Economically dependent on welfare benefits, she was obliged to comply with the directives. At the same time, as a single parent, Ms Reisch had to organize childcare for her child during her absence at rehabilitation, which was to be 8 weeks beginning at short notice, depending on the capacity of the rehabilitation provider. The solution intended by the guardian, in agreement with the public Youth Welfare Office (*Jugendamt*), was a household assistant (*Haushaltshilfe*), who would visit the then 12-year-old at home every day in the afternoon to support the child in housekeeping and doing homework for school. Ms Reisch was informed by the Youth Welfare Office that they did not provide foster families for children aged 12 and over. Due to the lack of a fixed starting date of the planned rehabilitation, registration of the child in one of the supervised shared flats was also not possible. The Employment Office’s broad time specification, that the rehabilitation stay would begin “in about 3 months”, was too vague for the Youth Welfare Office. Not only was the idea that her child should manage everyday life on its own for a few weeks unacceptable to Ms Reisch, it also deeply contradicted her vision of appropriate care. As a consequence, Ms Reisch entered a severe psychological crisis. Despite letters of appeal by the child’s psychologist, Ms Reisch’s psychologist, and the family doctor, the Employment Office was unwilling to postpone the measure to give Ms Reisch enough time to prepare for proper childcare. During our encounter, Ms Reisch explained this intricate and straining situation to me bluntly: “I have to go to rehabilitation in three months’ time. Whether I want to or not. And if I don’t want to go, my benefits will be cut off. Tough as nails (*ganz knallhart*).”^4^ I interpret this statement as an expression of feeling oneself at the mercy of others, in this case, the authorities.

Ms Reisch’s situation reveals several relevant social categories of inequality and so-called determinants of mental health: female, middle-aged, welfare beneficiary, single parent, mental illness, mental disability, and guardianship. These overlap clearly with the core welfare categories that define Ms Reisch’s situation according to social policy, which are ‘disability’, ‘mental illness’, and ‘single parenthood’. Given the concurrence of many of the social categories attributable to Ms Reisch, according to psychiatric health services research in the German national context, bad prognoses for mental health seem statistically probable [9]. Ms Reisch’s adverse situation, however, requires not confusing social factors with social causes.^5^ Rather, the case of compulsory rehabilitation indicates an analysis of *the doings* of social categories—both in the social sciences and in social policy. To this end, I first provide a brief introduction to the sociology of inequality, which is closely linked to the formation of welfare categories in the German welfare state, to then use a praxeographic approach to reassess the linkages between social theories on inequality and social policy, as well as the effects of these linkages.

### The sociology of social inequality

Since the sociological theory of social inequality develops concepts that describe structures of social organization and their effect on individual agencies, it is often referred to as social structure analysis. In the tradition of German sociology, the most traditional concepts are class and stratification theories, based on the work of the German philosophers Karl Marx and Max Weber. While Marx’s theory of society focuses on class conflict between the ruling and the working classes, capital concentration versus sparsity, and societal progress through revolutions, Weber provides a multidimensional social theory on class, status, and party that allows a more differentiated view of the formation of power in the state and the formation of collective identities. Even though Weber’s approaches allow for a much greater differentiation of social inequality than Marx’s, criticisms voiced since the 1980s concern not only the assumptions of both classical theories but also many of those who came after in inequality research [11]: (1) Instead of presupposing a gainful employment society and determining class and stratum membership according to the individual’s position in the gainful employment process, the sociology of inequality should pay close attention to changes in employment and income conditions and their interrelation with other social factors. (2) Increasing effects of globalization and internationalization need to be taken into account, and societies should not continue to be defined nationally. (3) In addition to vertical inequality expressed in dichotomies such as social up/down, social lower/higher or in the geologically informed metaphor of social layers, ‘horizontal’ differentiations need to be acknowledged. For instance, phenomena such as social difference with equal income or different pay for the same work should be included in the analysis.

Responding to this criticism and also to the classical class and stratum model, inequality research in recent years has provided a number of additional concepts to describe the structure of society and responses to social change. These include theories on lifestyles, milieus, or social positions. All these still have in common that they need to meet the challenge of defining *relevant* social characteristics for analysis and decide on their significance [12]. Across theoretical models, inequality research defines social inequality as:

“a social state in which access to important social areas (e.g. education and training, occupation) is more difficult for individuals or social groups and the unequal distribution of economic and other resources, of social positions and ranks is seen as a social problem. Social positions and ranks that are assessed as unequal are associated with different ways of exercising power and domination and the appropriation of resources.” [13]

This definition emphasizes that both different access to different social areas for individuals and groups and the unequal distribution of different resources exist as a *social* rather than an individual problem. Accordingly, social inequality is strongly related to social in*justice*; otherwise, there would be no social problem. This is also evident in the second sentence, which makes clear that in this sense unequally valued positions mean different access to resources and power. I build on this definition and retain the historically established analytical sensibilities of the concept of social inequality, adding, from a praxeographic angle, the perspective that inequality, rather than a social *state*, also is an effect of situated processes that bring about differentiations of equal and unequal. This praxeographic approach to in/equality foregrounds how equality and inequality are produced in practice. By introducing the slash in the spelling of the term in/equality, and related word forms, I want to emphasize the ambivalences of the practices under study: Sorting people, groups, or social positions, in social theory as much as in social policy, always means enacting equalities (sameness) and inequalities (difference) in tandem. It is the ambiguity and the consequences of this sorting that need to be determined empirically, especially if we want to know more about how social policies contribute to making people and social conditions un/equal.

### Creating in/equality

To attempt such a praxeographical study of in/equality on the empirical case of the prescribed rehabilitation measure described above, I take up the research program on human differentiation as developed by the German sociologist Stefan Hirschauer [8]. In the past decade, Hirschauer and his team had been increasingly concerned with the social relevance of categorical differentiation. Their research program, “Un/doing differences: Practices of human differentiation”, focuses on the production of social *belonging* through the classification of people (foreign and self-attributed). The analytical connection between equality and inequality with practices of comparison is the basis for this research program: It is through comparison that sameness or difference are socially created. Through comparison, we state what we consider equal or unequal, or we compare to create what is later to be considered equal or unequal. In conclusion, in/equality is to be studied primarily as a practice of distinguishing.

Hirschauer refers to the following two characteristics of social differentiations [8]: (1) Differentiations are qualitatively very heterogeneous, differing in terms of their distinguishing features (e.g., body, performance, and gender) and the social forms they produce (e.g., individuality, communities, and movements). (2) Defined groups differ in the ‘intensities’ in which members (may) belong to them. For example, citizenship can be a very rigid membership, while memberships in leisure groups can be much more flexible, and some memberships can also be or become irrelevant to the lifeworld. Here, Hirschauer identifies the danger that social science research might convert group memberships into social characteristics of individuals (e.g., considering someone disabled because of a disability card) instead of seeing the categories themselves (e.g., disability vs. ability) as characteristics of the organization of society [8].

According to Hirschauer, these latter characteristics of social organization should be the focus of sociological research. He pursues this by taking the categorisation of differences (i.e., inequalities) itself as the object of research and calling for a stronger “cultural-sociological line of research of a theorisation of human differentiations” [8]. The research program, therefore, focuses on multiple belonging, hence on the question: “What difference is in effect where and when?” [8]. This requires, on the one hand, relativizing difference (what difference?) and, on the other hand, temporalizing it (when?):

“There is therefore a need for studies on cultural human differentiations, which start out from their mutual relativization, reflexively observe one’s own use of differentiation (namely on the side of the researchers) and systematically reckon with the fact that every differentiation can also be overlaid by other differentiations, lose relevance and disappear.” [8]

According to this program, social belonging and differences are practically done—or practically rendered ineffective. This is expressed by the phrase “doing and undoing difference” [8].^6^ Recalling the definition of social inequality as provided by the sociology of inequality, Hirschauer’s definition of human differentiations brings into focus social processes that produce distinctions between equal and unequal, thus creating this very differentiation.

In this sense, the approach of un/doing differences urges caution about the extent to which social structure analyses or the social determinants of mental health framework convert psychiatric diagnoses and other ‘social’ factors, such as employment and parenthood into individual or social characteristics and how they impact social policies. While knowledge of such numbers and figures is clearly extremely important for the knowledge about society, I argue that these results need to be complemented by knowledge about what mental health and illness come to mean to people in their everyday lives and how mental health and social policies, designed to alleviate social inequalities, are experienced. This is especially important if we want to change how social services implement and undo differences and thus participate in creating these experiences. In this respect, the empirical case of the mandatory rehabilitation stay is highly instructive. In the continuing analysis of the case, I regard welfare categorization processes as part of what the sociologist Michael Schillmeier terms “the experience and the event of dis/ability” [15]. In Schillmeier’s approach, dis/ability becomes a heterogeneous object to which both the body—with its psychological and physical forms of expression—and social ways of ordering belong. Schillmeier thus synthesizes the medical and the social model of disability and foregrounds the relations between the ‘natural’ and the ‘cultural’ that constitute dis/ability. Independently, but in line with Hirschauer’s research program, Schillmeier proposes conceptualizing dis/ability as an enactment of difference that is physically located and socially practiced at the very same time. Mental ill health is frequently related to disability conceptually, not least in and through the available welfare registers [16–18].

### Attending to the experiences and events of dis/ability

Returning to the case of the prescribed rehabilitation stay against the theoretical background of a praxeographically informed sociology of in/equality, more information is needed to understand how Ms Reisch’s situation came about and how certain particular differentiations have been made relevant and played out. My analysis is as follows: (1) Ms Reisch had been allocated a legal guardian by the German state, because she was classified as ‘mentally ill’ due to a psychiatric diagnosis. Because of the diagnosis, she was entitled to publicly funded medical and psychological appointments. (2) Because Ms Reisch held the legal status of ‘severe disablement’, following the period of sickness benefit, the Employment Agency paid her a ‘transitional allowance’ (*Übergangsgeld*).^7^ That was why it was the responsible authority for proposing rehabilitation measures. (3) In 2013, Ms Reisch was a single parent of one child, aged 12. Since Ms Reisch had started psychiatric treatment, the Youth Welfare Office supported her with childcare. The age of the child is important for the Youth Welfare Office’s approach as offers of support are conveyed in different ways depending on age. (4) Ms Reisch had no savings so was dependent on social benefits (in her case the payments of the Employment Agency) and on state support for the care of her child.

The prescription of the medical rehabilitation measure, which was intended to contribute to Ms Reisch’s psychological recovery and her re-integration in the employment process, created a situation in which Ms Reisch was significantly restricted in her responsibility as a parent. Rather than rejecting psychosocial rehabilitation in principle, she was concerned with making responsible decisions for her child. Since she was financially dependent on the public authorities, the particular practice of prescribing rehabilitation prevented her from doing so. In short, the administrative order led to psychological recovery and parenthood being played off against each other. Under the close supervision of her psychologist and with the help of friends, who took in the child without any ado, Ms Reisch finally overcame the psychological strains triggered by the prescription and managed to set up a private solution among friends for good childcare that made the rehabilitation stay possible. As a social service for support of those classified as ‘severely disabled’ and ‘mentally ill’, the rehabilitation directive had worked directly against the interests and health of Ms Reisch as a legally entitled person and her child.^8^ It was only because she succeeded in mobilizing divergent forces (conventionally referred to as psychological, physical, or social) that was she able to realize a life worth living for herself and her child despite (not due to) the prescribed welfare measure, and to escape another acute admission to the psychiatric hospital—an experience that, by her own account, had previously marked the lowest point in her life.

While welfare statistics of mental well-being would presumably miss Ms Reisch’s psychological crisis, because she skilfully avoided a hospital admission and, from the authorities’ point of view, appeared at rehabilitation as scheduled, this praxeographic analysis highlights how, beyond the radar of statistics, social services co-create the lifeworlds in which mental health and breakdown are experienced. In other words, praxeography enables the investigation of the unexpected effects of the welfare system that would escape the data of traditional analysis of social inequality and social determinants. Ms Reisch’s strained situation is not the outcome of an exceptional case of mismanagement in social services. Given that overlapping of multiple welfare categorisation processes as in Ms Reisch’s case is common rather than exceptional, this empirical analysis underlines the need for understanding the quandaries that established welfare practices create. In Ms Reisch’s case, social services for rehabilitation of people classified as ‘mentally disabled’ had restricted her in parenthood: The practices organized by the differentiations of ‘severely disabled’, ‘mentally ill’, and ‘single parent’ in the German welfare framework led to effects that ultimately clearly run counter to the stated policy intentions. At the same time, social services for those classified as ‘mentally ill’, such as publicly funded appointments with psychologists, supported Ms Reisch in crafting a tailor-made solution *outside* the welfare system for a situation she should never have got into.

### Working toward post-categorical social policy

If practices of social welfare co-produce social in/equality and dis/ability, we must not only study these practices and explore how they affect people adversely, but also consider ways to change them. As the investigation into Ms Reisch’s situation demonstrates, social categories substantially orchestrate welfare practices. It is multiple classification processes that allocate social services to people and hence shape their experiences. At the same time, as the philosopher Ian Hacking has demonstrated, experiences impact classification processes [20–23]. It seems that, to *loosen* the attachment of categories *to people* in public administration and to avoid potentially self-fulfilling circular reasoning when addressing social crises through established formal and informal essentializing categories *of people*, this process of mutual enactment requires improvement. Administration should accordingly be supported in its ability to respond to events and experiences of dis/ability as they evolve in practice. Social sciences have long demonstrated that reflection and learning do not happen in human minds or brains in any isolation, but unfold in socio-material relations and the practical engagement with the world [24, 25]. Consequently, implementing socio-material infrastructures of welfare that incorporate “response-ability” [26], that is the ability to reflect on unfolding developments and adapt accordingly, seems imperative.

Reflections on such infrastructures inspired my study of the German Social Code as the legal foundation of social policy and ultimately led me to post-structuralist legal studies. By propagating the option of a *post-categorial* antidiscrimination law, the field of post-structuralist legal studies strives to avoid law reifying social conditions of inequality or essentialising concepts of group identities [27]. With regard to dis/ability, this legal approach focuses on the prohibition of ableist discrimination and omits the currently legally customary assessment of “whether physical, mental and psychological characteristics constitute a disability” [28] on an individual level. This approach thus decentres the differentiation of dis/ability and demands of legal procedures to inquire into the production of discriminatory experiences that result from ableist practices. Accordingly, the Social Code could define ableist *processes* as crucial classification units in social policy rather than questionable characteristics of people, such as disability. The crucial innovation here would be that, as a consequence, the target of policy measures shifted from the person ‘with disabilities’ toward the decision-making institutions, such as public authorities or private enterprises, who would be legally under obligation to break down ableist practices [28]. For Ms Reisch, a post-categorical approach to social policy would have meant that social services inquired into ableist practices that constituted the events and experiences of dis/ability in her case and determined what countermeasures the authorities and her employer were obliged to take to change the situation. Since welfare practices have the potential to largely shape the available ambiguous opportunities for being-in-the-world un/equally, future studies should explore how social theory can contribute to enumerating the possibilities, effects, and implications of a post-categorical social policy framework.

## Data Availability

Data and material generated in ethnographic research are always socially embedded and co-produced by researchers and research partners, which makes data-sharing a particular ethical concern. Ethnographic data can only be understood in context and might reveal sensitive information about sites and people, even if researchers anonymize it. For further information, see the Position paper issued by the German Anthropological Association (GAA) on the handling of anthropological research data (2019). Ethical approval for this study was obtained before policies on the availability of ethnographic research data were started to be established. Research partners’ consent to this study therefore did not include the open archiving and sharing of data. In view of these ethical considerations, ethnographic data supporting this study are not publicly available.

## Footnotes

1 Yates-Doerr (2020) also makes the case for a material-semiotic approach to health and brilliantly details how this approach differs from the social determinants of health framework [7].

2 For project details, see the acknowledgements section.

3 The name is a given pseudonym for the purposes of research.

4 In this paper, all translations from German are mine.

5 For a detailed proposal of a “new ‘mechanism-rich’ epidemiology” that goes beyond social factors and describes *mechanisms* as relations responsible for biosocial phenomena, see Manning 2019 [10].

6 Hirschauer builds on West’s and Zimmerman’s (1987) famous approach of “doing gender” [14].

7 Ms Reisch was entitled to sickness benefits (rather than unemployment benefits), because she had been employed when entering psychiatric hospital treatment.

8 For a thoughtful ethnographic analysis of ambiguous effects of state policies on psychiatric care users in Italy, see Giordano (2018) [19].

## References

[1] Silva M, Loureiro A, Cardoso G (2016). Social determinants of mental health: a review of the evidence. Eur J Psychiatry.

[2] Johnson S (2017). Social interventions in mental health: a call to action. Soc Psychiatry Psychiatr Epidemiol.

[3] Zacher HF (2014). Social Policy in the Federal Republic of Germany: the Constitution of the Social.

[4] Mol A (2002). The Body Multiple. Ontology in Medical Practice.

[5] Latour B (2005). Reassembling the Social. An Introduction to Actor-Network-Theory.

[6] Latour B (2004). Why has critique run out of steam? From matters of fact to matters of concern. Crit Inq.

[7] Yates-Doerr E (2020). Reworking the social determinants of health: responding to material-semiotic indeterminacy in public health interventions. Med Anthropol Q.

[8] Hirschauer S (2017) Un/doing Differences: Praktiken der Humandifferenzierung. Velbrück Wissenschaft, Weilerswist

[9] Jacobi F, Höfler M, Siegert J (2014). Twelve-month prevalence, comorbidity and correlates of mental disorders in Germany: the Mental Health Module of the German Health Interview and Examination Survey for Adults (DEGS1-MH). Int J Methods Psychiatr Res.

[10] Manning N (2019). Sociology, biology and mechanisms in urban mental health. Soc Theory Health.

[11] Kreckel R (1983). Soziale Ungleichheiten.

[12] Burzan N (2011). Soziale Ungleichheit: Eine Einführung in die zentralen Theorien.

[13] Schäfers B, Lehmann B, Kopp J, Schäfers B (2010). Soziale Ungleichheit. Grundbegriffe der Soziologie.

[14] West C, Zimmerman DH (1987). Doing gender. Gend Soc.

[15] Schillmeier M, Schneider W, Waldschmidt A (2007). Zur Politik des Behindert-Werdens. Behinderung als Erfahrung und Ereignis. Disability Studies, Kultursoziologie und Soziologie der Behinderung: Erkundungen in einem neuen Forschungsfeld.

[16] Bister MD (2018). The concept of chronicity in action: everyday classification practices and the shaping of mental health care. Sociol Health Illn.

[17] Bister MD (2020). Gesundheitliches Gut-achten als soziomaterieller Prozess: Eine Praxeografie der Zuerkennung von gemeindepsychiatrischen Leistungen in Berlin. Österr Z Für Geschichtswissenschaften.

[18] Bister MD, Svalastog AL, Gajovic S, Webster A (2021). Minute/s work: the participation of digital data objects in the conjuncture and disjuncture of policy and care. Navigating digital health landscapes: a multidisciplinary analysis.

[19] Giordano C (2018). Political therapeutics: dialogues and frictions around care and cure. Med Anthropol.

[20] Hacking I (2007). Kinds of people: moving targets. Proc Br Acad.

[21] Bieler P, Bister MD, Niewöhner J (2023) Phänomenographie: Zur Rekonstruktion von Erfahrung als Praxis. In Röthl M, Sieferle M (eds) Erfahrung: Kulturanalytische Relationierungen—Positionen und Zugänge der Europäischen Ethnologie. Waxmann, Münster, pp 59–78

[22] Bieler P (2021) BioÖkologien des Begegnens: Eine ethnografische Untersuchung der relationalen Konstitution psychischer Gesundheit und urbaner Umwelten. Unpublished PhD Thesis, Humboldt-Universität zu Berlin

[23] Bister MD, Klausner M, Niewöhner J, Blok A, Farías I (2016). The cosmopolitics of, niching’. Rendering the city habitable along infrastructures of mental health care. Urban cosmopolitics: agencements, assemblies, atmospheres.

[24] Bateson G (1972) Steps to an Ecology of Mind. Collected Essays in Anthropology, Psychiatry, Evolution and Epistemology. University of Chicago Press, Chicago

[25] Ingold T (2011). Being alive: essays on movement, knowledge and description.

[26] Haraway D (2016). Staying with the trouble: making Kin in the Chthulucene.

[27] Baer S (2010) Chancen und Risiken Positiver Maßnahmen: Grundprobleme des Antidiskriminierungsrechts und drei Orientierungen für die Zukunft. In Heinrich Böll Stiftung (ed) Positive Maßnahmen—Von Antidiskriminierung zu Diversity. https://heimatkunde.boell.de/sites/default/files/dossier_positive_massnahmen.pdf Accessed July 2023

[28] Liebscher D, Naguib T, Plümecke T, Remus J (2012). Wege aus der Essentialismusfalle. Überlegungen zu einem postkategorialen Antidiskriminierungsrecht. Krit Justiz.

